# The impact of childhood glaucoma on psychosocial functioning and quality of life: a review of the literature

**DOI:** 10.1038/s41433-023-02492-1

**Published:** 2023-03-22

**Authors:** Danielle E. McLaughlin, Ana Semrov, Hounsh Munshi, Annika J. Patel, Jugnoo Rahi, Alana L. Grajewski, Balkan CGRN Study Group, Elena Bitrian, James D. Brandt, Ta Chen Chang, Tanuj Dada, Alan Delamater, Beth Edmunds, Sharon F. Freedman, Elizabeth Hodapp, Youngmee Kim

**Affiliations:** 1 Bascom Palmer Eye Institute, University of Miami Miller School of Medicine, Miami, FL USA; 2 Population, Policy and Practice Research and Teaching Department, GOS Institute of Child Health, University College London, London, UK; 3 Ulverscroft Vision Research Group UCL, London, UK; 4 Great Ormond Street Hospital NHS Foundation, London, UK; 5 Institute of Ophthalmology UCL, London, UK; 6 Department of Ophthalmology, University of California, Davis, Sacramento, CA USA; 7 RP Centre for Ophthalmic Sciences, All India Institute of Medical Sciences, New Delhi, India; 8 Department of Pediatrics, University of Miami, Miami, FL USA; 9 Casey Eye Institute, Department of Ophthalmology, Oregon Health & Science University, Portland, OR USA; 10 Department of Ophthalmology, Duke University Medical Center, Durham, NC USA; 11 Department of Psychology, University of Miami, Coral Gables, FL USA

**Keywords:** Education, Quality of life

## Abstract

We present a novel comprehensive literature review of studies of the psychosocial functioning (PF) and quality of life (QoL) of patients with childhood glaucoma and their caregivers. Our findings demonstrate variable study quality and approach, as well as inconsistent results relating to the association of glaucoma-specific factors and sociodemographic variables with measured PF and QoL. Future studies should focus on the development of culturally cognizant and standardized assessment tools, execution of multi-center longitudinal studies with global representation, evaluation of PF and QoL among siblings and childhood glaucoma providers, and implementation of interventions to improve patient and caregiver PF and QoL.

## Abstract (zh)

我们对儿童青光眼患者和其监护人的心理功能 (PF) 和生活质量 (QoL) 进行了全新以及具有深度的文献回顾。研究结果表明, 该方面研究的质量和方法各不相同, 而且关于青光眼特定因素和社会人口变量与测定的PF和QoL之间的关系的结果也不一致。未来的研究应集中于开发具有文化认知并经过标准化的评估工具, 开展具有全球代表性的多中心纵向研究, 评估兄弟姐妹和儿童青光眼病例提供者的PF和QoL, 以及实施干预措施来改善患者及监护人的PF和QoL。

## Introduction

While literature exists on the effects of childhood glaucoma on psychosocial functioning (PF) and quality of life (QoL) from the perspective of the patient or family, there is no consensus on the magnitude of the impact of this diagnosis on patients and family members. Extrapolations from studies on other pediatric vision disorders [1–9] and chronic pediatric conditions [10–13] imply that childhood glaucoma may have significant outcomes on the QoL of the patient, caregivers, and siblings [14]. Patient-reported outcome measures (PROMs) allow clinicians to better understand the patient’s perception of their own well-being and functioning with regard to their disease [15]. Increased clinical implementation of PROMs and other psychosocial tools in recent years has revealed an association between pediatric patient or caregiver QoL and treatment adherence and disease outcome [16–19]. We review the literature regarding PF and QoL in childhood glaucoma, and the tools available for their assessment. This review will provide a synthesis of existing literature, highlight the relevance of these factors in the holistic care of patients with glaucoma and their families, and emphasize the importance of including this type of data in studies of clinical and surgical outcomes in childhood glaucoma. We hope to motivate further research in this area with the goal of improving overall health outcomes for patients with childhood glaucoma and their caregivers.

### Childhood glaucoma

Childhood or pediatric glaucoma (henceforth referred to as “childhood glaucoma”) is classified into two diagnostic categories: primary glaucoma and secondary glaucoma [20]. Primary glaucoma is not associated with other ocular or systemic diseases and is classified by age of onset into primary congenital glaucoma (PCG) and juvenile open-angle glaucoma (JOAG). Secondary glaucoma may be associated with either a non-acquired systemic disease, syndrome, or ocular anomaly or with acquired conditions such as trauma or inflammation. A separate category exists for glaucoma following cataract surgery [20, 21]. In this review, “childhood glaucoma” will encompass all childhood glaucoma diagnoses.

#### Prevalence and incidence

Due to a lack of data, an accurate estimate of the global prevalence or incidence of childhood glaucoma cannot be determined. Ethnicity and consanguinity are believed to influence rates of glaucoma [22], further complicating the global approximation of cases. Glaucoma accounts for 0–7% of pediatric blindness depending on the region [23, 24], and it is estimated that one in 10,000 babies in the United States is born with PCG [25], and that a general ophthalmologist may encounter one new case of PCG every five years [26, 27]. In one U.S. county with an incidence of 2.29 cases of childhood glaucoma per 100,000 residents under age 20, most cases were secondary, either non-acquired or acquired, while PCG and JOAG were rare [28].

#### Health outcomes

Childhood glaucoma is characterized by elevated intraocular pressure (IOP) and its effects on the structures of the young eye. These will vary depending on the age of onset and the severity and duration of the elevated IOP. If left untreated, elevated pressure damages the optic nerve, leading to loss of the optic nerve fibers and producing “cupping” of the nerve head; these structural changes manifest functionally as progressive and irreversible vision loss including blindness [29]. Other clinical features include progressive myopia, photosensitivity, eye enlargement (buphthalmos), watery eyes (epiphora), and cloudiness (corneal edema). Thus, not only does childhood glaucoma cause visual disability, it can also involve cosmetic changes which may impact the patient’s emotional adjustment or self-esteem as has been reported in pediatric cases such as skin disorders [30, 31] and Marfan syndrome [32].

Because childhood glaucoma is rare with sometimes subtle or unfamiliar clinical presentations, it can be diagnosed late or misdiagnosed altogether [26]. In many areas, access to ophthalmologists with training or expertise in childhood glaucoma is limited, compounding delays in diagnosis and treatment [26, 33]. In addition to negative ocular health outcomes, childhood glaucoma and its associated management and follow-up can significantly impact the patient and immediate family members’ PF and QoL, which will be the focus of the rest of the review.

### Defining key concepts

#### Psychosocial functioning

Although the term lends itself to variable interpretations, PF is defined by Ro and Clark according to four domains: well-being, basic functioning, self-mastery, and interpersonal and social relationships. In general, it is the ability of an individual to engage in daily activity, partake in societal roles, and develop social relationships.

Well-being refers to an individual’s life satisfaction and self-acceptance while basic functioning encompasses mobility, participation in society, and physical ability. Self-mastery relates to internal self-control, and lastly, interpersonal, and social relationships pertain to empathy and agreeableness [34].

#### Quality of life

QoL is an individual’s subjective perception of life in the context of their culture, society, environment, and expectations, as defined by the World Health Organization, and encompasses physical, psychological, and social health [35]. Studies sometimes refer to QoL as it pertains to vision in three ways: health-related (HR-QoL) [36, 37], vision-related (VR-QoL) [38], and functional vision (FV) [36] (also referred to as functional visual ability or visual functioning). However, it is important to note that these are distinct, though related, constructs.

HR-QoL is the subset of QoL and refers to the individual’s perception of their physical or mental health status [39, 40]. VR-QoL, similar to HR-QoL, defines an individual’s perception of their well-being as it pertains to their vision [38, 41]. FV is a different and distinct concept that measures an individual’s visual ability to perform daily activities or tasks [42, 43], rather than the impact of the individual’s vision on their well-being, which is assessed by QoL measures. Unfortunately, many studies and published instruments conflate QoL and FV [44].

#### Patient-reported outcome measures

PROMs assess the patient’s own perception of their status with respect to their health or specific diagnosis and can measure any of the following different concepts: HR-QoL, functional status, symptoms, or health behavior [45].

#### Caregiver

Caregiver encompasses both formal (i.e., ophthalmologist) and informal caregivers (i.e., parents and siblings) and refers to any person who helps an individual with childhood glaucoma with their disease management or activities of daily living [46].

Because the current review encompasses no study involving caregivers other than parents, “caregivers” hereafter solely describes parents of individuals with childhood glaucoma.

Most relevant to this review is the caregiver burden; the multidimensional, self-perceived strain a person may feel for caring for their family member, loved one, or patient over time [47].

### Disease and psychosocial function/quality of life

#### Relationship between chronic pediatric conditions and PF or QoL

The effects of the physical limitations and treatment demands from various chronic pediatric conditions extend beyond the patient’s physical health status. For instance, children and young people with chronic health conditions are at higher risk for mental health problems (e.g., anxiety and depression), and psychosocial, behavioral, and academic impairment as compared to their peers without the chronic disease [10, 11, 48–51].

From an early age, children with chronic disease depend on their caregivers for support with treatment, medical appointments, and activities of daily living, resulting in caregivers experiencing physical and mental health complications [52–56], extensive time demands [55], financial burden [55, 57], family stress or patient-caregiver tension [58], and low QoL [59, 60]. The caregivers’ QoL may impact the care they provide, affecting patient medication adherence and, in turn, disease outcome, patient QoL, and psychosocial adjustment [16, 18, 19]. Everhart et al. [16] report that this relationship can operate in the reverse direction as well, where improved medication adherence results in the improved health status of the child, minimizing caregiver anxiety.

#### Disease factors of childhood glaucoma that may affect PF or QoL

Childhood glaucoma and visual impairment may physically limit the patient, restricting their academic, social, and extracurricular activities. It is a lifelong disease demanding routine visits or procedures with a physician that, extrapolating from chronic pediatric diseases previously mentioned, may impose a financial, timewise, and personal opportunity cost for the patient, caregivers, and family unit [14]. Almost all cases of childhood glaucoma require chronic eye drop use, prescription glasses, and/or surgery, with more than a third (39.4%) needing more than one procedure [61]. Prescription glasses negatively affect children’s self-esteem [62] and administering eye drops in pediatric patients may be distressing for the child and caregiver because of discomfort and patient resistance [62, 63], hindering therapy compliance and potentially impacting disease progression. Such progression leads to impaired vision, which studies link to higher levels of depression [64–66] and a greater independent risk factor for suicide [67] than neurological disorders and malignant disease [68].

Furthermore, the progressive nature of vision disorders such as glaucoma leads to illness and prognosis uncertainty, which correlates positively with patient depression and anxiety [69]. Although limited literature exists on this relationship for caregivers of individuals with vision disorders, studies performed on other pediatric conditions report a negative impact of illness uncertainty on caregiver well-being [58, 70]. This risk can be mitigated by improving health literacy (e.g., knowledge and expectations of disease, treatment, or other health factors) for patients and caregivers, which decreases patient anxiety [71] and improves medication adherence [72]. By enhancing patient and caregiver understanding of childhood glaucoma, individuals are more likely to comply with treatment, potentially improving their physical and mental health outcomes.

By reviewing the current literature and exploring assessment tools available on childhood glaucoma, the authors hope to encourage PF and QoL support initiatives in an effort to improve childhood glaucoma outcomes.

## Method of literature search

### Database and search terms

The authors performed the initial literature search through the PubMed database in August 2021 and repeated the search twice more (in March and April 2022). One author (DEM) performed the database search and screened papers by inclusion criteria, which was later verified by two subsequent authors (AS and HM). The authors used the following combinations of search terms or closely related ones: “Childhood glaucoma OR pediatric glaucoma OR glaucoma” AND “quality of life OR life experience OR caregiver burden OR mental health OR psychology OR psychosocial OR family psychology OR cognitive development OR academic function”.

The authors conducted the search in two stages: once without an age filter to capture caregivers, parents, immediate family member participants, and adults who were diagnosed with glaucoma at a pediatric age and a second time with an age filter to include only newborn, infant, preschool child, child, and adolescent patients. For both stages, authors screened articles by topic based on their titles or abstract. The authors excluded papers pertaining to eye diseases other than glaucoma or papers on topics unrelated to PF or QoL. The search with the age filter did not contribute any articles not previously captured by the unfiltered search.

### Inclusion and exclusion criteria

The authors used the following inclusion criteria when selecting literature: (1) original abstracts available in English; (2) research studies of any methodology; (3) studies from any country of publication; (4) studies from any year of publication; (5) study participants who were diagnosed with glaucoma at a pediatric age or study participants who were considered to be parent, caregiver, or immediate family member of a patient with childhood glaucoma; and (6) studies on the topic of PF or QoL. The authors excluded literature if (1) the paper was on any disease not relevant to childhood glaucoma, (2) the paper was on any topic not relevant to PF or QoL, and (3) it was a literature review and/or meta-analysis paper.

All identified papers included an English language abstract. The authors used Google Translate for papers with an English abstract and non-English articles.

## Results

**Fig. 1:**
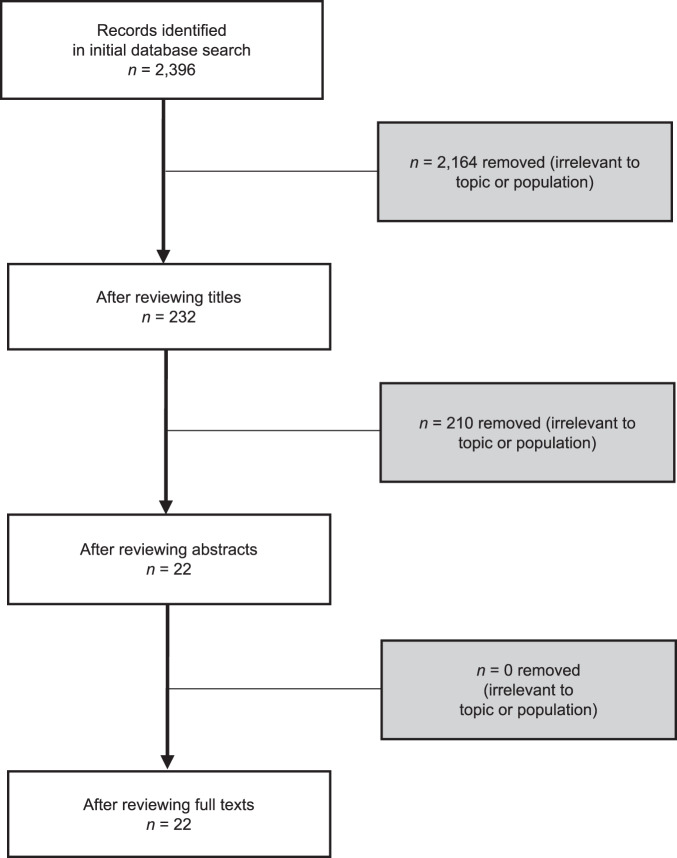
Flow diagram of study selection. This outlines the number of papers included in the review (“*n*”) after screening sequentially by titles, abstracts, and full texts (white boxes on the left). It also includes the number of papers removed (“*n*”) due to an irrelevant topic or population identified through each screening step (grey boxes on the right).

The initial database search yielded 2396 papers (Fig. 1) and, following the inclusion and exclusion criteria previously outlined, the database search resulted in the identification of 22 papers.

Out of 22 papers, 2 [73, 74] focused solely on questionnaire development and validation, while the remaining 20 measured aspects of PF and QoL in patients with childhood glaucoma and their caregivers with two studies also assessing the psychometric properties of the questionnaire used [37, 75]. Alternatively, one study measured the agreement between the patients’ and caregivers’ report of the patient’s HR-QoL [76]. Most studies (*n* = 13) focused on children and young people with glaucoma [36–38, 71, 75–82], while others (*n* = 9) studied caregivers of these patients [38, 73, 83–89].

Many of the included papers investigated associations between the main outcomes (i.e., QoL or FV) and at least one clinical, treatment, or disease-specific factor. For instance, the studies most frequently compared PF or QoL findings to glaucoma type or laterality [36–38, 75, 77, 78, 83, 85, 88, 90], visual acuity (VA) [36–38, 75, 77–79, 82, 83, 88, 90], age at or duration since diagnosis [36, 38, 77, 79, 83, 86, 87], medication or treatment practices [36, 71, 75, 77–79, 81–83, 88], and number and outcomes of glaucoma surgeries [36, 38, 75, 77–79, 81–83, 85–88]. In relation to family and broader sociodemographic characteristics, researchers most frequently compared outcome measures to age, gender, marital status, parental education level, employment status, household income, socioeconomic status, and presence of another child with glaucoma in the family [36–38, 75, 77–79, 81, 83, 85–88, 90].

The findings of the present literature review are discussed from the perspective of patients with childhood glaucoma and their caregivers.

### The impact of childhood glaucoma on patients

The summary of studies on patients with childhood glaucoma is outlined in Table 1, with correlates and predictors of QoL and/or FV presented in Table 2. Most studies investigated associations between different clinical and sociodemographic factors and QoL and/or FV, with only one study measuring other factors.

**Table 1:** Summary of the studies on patients with childhood glaucoma.

Author, publication year, and country	Study aim	Study type and area of interest	Sample size	Age range; average age	Instruments and reporter	Data analysis	Main results
AlDarrab et al. (2019) Saudi Arabia	Assess FV and VR-QoL of children with glaucoma	Cross-sectional (questionnaire) FV, VR-QoL	85 children with glaucoma	8–18 years; *M* = 14 years	CVAQC IVI-C Self-report	Correlations, analysis of between-group difference	Worse FV: worse BVCA, bilateral glaucoma, ≥3 glaucoma surgeries, and more glaucoma daily eye drops bilaterally; Not associated with child’s age, age at diagnosis, type of glaucoma, parental educational level, number of children with glaucoma in the family Worse VR-QoL: worse BVCA, bilateral glaucoma, ≥3 glaucoma surgeries, children with illiterate parents; not associated with child’s age, age at diagnosis, type of glaucoma, number of glaucoma daily eye drops bilaterally
Dahlmann-Noor et al. (2017) UK	Assess FV, VR-QoL, HR-QoL of children with glaucoma	Cross-sectional (questionnaire) FV, VR-QoL, HR-QoL	119 children with glaucoma and their parents	2–16 years; *M* = 9.4 years	CVAQC IVI-C PedsQL Self-report and parent-/family- report	Correlations, 2-sample Wilcoxon rank-sum or Mann–Whitney *U* test, Bland–Altman techniques	Worse FV: worse BCVA, decreasing age, bilateral glaucoma Worse VR-QoL: worse BCVA, decreasing age; not associated with glaucoma laterality Worse HR-QoL: worse BCVA; not associated with child’s age; children with bilateral glaucoma had worse HR-QoL than children with unilateral glaucoma in case of a parent- but not in case of self-report
Freedman et al. (2014) USA	Assess self-reported VR-QoL of children with glaucoma	Cross-sectional (questionnaire) VR-QoL	43 children with glaucoma	5–17 years; *M* = 11 years	IVI-C Self-report	*t*-test, ANOVA, RA	Linear RA: better VR-QoL scores were associated with better BCVA in all types of glaucoma and in subgroup of patients with bilateral glaucoma; not associated with sex, age, race, laterality of glaucoma, number of eye surgeries, number of glaucoma medications prescribed, and the frequency of prescribed dosing
Gothwal and Mandal (2021) India	Evaluate QoL, life satisfaction, and their predictors in young adults treated for PCG during early childhood	Cross-sectional (questionnaire) generic QoL, satisfaction with life	82 emerging and young adults with PCG	18–34 years; *M* = 22.5 years	WHOQOL-BREF SWLS Self-report	*χ*^2^ statistic for proportions, *t-*test, RA, one-way ANOVA, correlations	Better QoL: more years of education and rural residency; not related to age, and other clinical/treatment-related variables Higher life satisfaction: unilateral glaucoma, more years of education, and being married; not related to age, and other clinical/treatment-related variables Multivariable RA: participants in rural residence and with more years of education reported better QoL (*R*^2^ = 13%); married participants reported better SWL (*R*^2^ = 8.8%); none of the clinical and treatment-related variables were important predictors of QoL/SWL scores
Gothwal et al. (2018) India	Measure parent–child agreement on child’s HR-QoL in children operated for PCG	Cross-sectional (questionnaire) HR-QoL	121 children with PCG and their parents	8–18 years; *M* = 11.8 years	Kidscreen-27 Self-report and parent report	RA, Bland–Altman LoAs	Bidirectional disagreement between parent–child HR-QoL reporting Discordance between children and parent reports was greater in case of younger children and girls
Gothwal et al. (2019) India	Assess HR-QoL of children operated on for PCG and identify sociodemographic factors associated with HR-QoL	Cross-sectional (questionnaire) HR-QoL	121 children with PCG	8–18 years; *M* = 11.8 years	Kidscreen-27 Self-report	RA, Cohen’s *d*	Multiple linear RA: lower HR-QoL was related to decreasing age (*R*^2^ = 23%); not associated with gender, duration since surgery, BCVA, glaucoma type or laterality
Gothwal et al. (2020) India	Compare FV and VR-QoL of children with PCG and children with SCG	Cross-sectional (questionnaire) FV, VR-QoL	309 children with treated PCG or SCG	8–18 years; *M* = 12.4 years	LVP-FVQ-II IVI-C Self-report	RA, Cohen’s *d*	Worse VR-QoL: SCG, worse VA in the best eye; not associated with age, sex, duration and laterality of glaucoma, and number of glaucoma surgeries Worse FV: SCG, worse VA in the best eye, bilateral glaucoma, >1 glaucoma surgery; not associated with age, sex, and duration of glaucoma Multiple linear RA: better VR-QoL was independently related to better VA and PCG; Better FV was independently related to better VA, unilateral glaucoma and PCG
Knight et al. (2021) Australia and New Zealand	Examine QoL issues of adults with childhood glaucoma to inform development of childhood glaucoma-specific PROM for adults	Qualitative (interview) PF and QoL	47 adults with childhood glaucoma	≥18 years; *M* = 40.0 years	Semi-structured interviews Self-report	Interpretive phenomenology	Ten major QoL themes were found: coping, emotional well-being, ocular health concern, symptoms, family planning, inconveniences, social well-being, activity limitation, economic, mobility
Miraftabi et al. (2020) Iran	Examine VR-QoL of adults with PCG	Cross-sectional (questionnaire) VR-QoL	23 adults with PCG	18–40 years; *M* = 29.2 years	VFQ-25 Self-report	Correlations	VR-QoL: increasing age was negatively associated with general vision, distance activity, and peripheral vision subscales; increasing visual field mean defect was positively associated with general health, general vision, near activity, distance activity, social functioning, mental health subscales and total QoL scale; not associated with BCVA, IOP, and number of surgeries
Mohamed et al. (2011) Eqypt	Assess the effect of an educational program on the knowledge and practices of glaucoma and eye care among adolescent patients with glaucoma	Longitudinal (questionnaire, intervention evaluation) Disease management and expectations, daily living skills, child’s mental health, child’s self-esteem	50 children with glaucoma	12–18 years; *M* = 15.9 years	Glaucoma knowledge Physical and social assessment CMAS CDI SEI Patients’ Expectations Scale Self-report	Frequencies, percentages, *χ*^2^ test, *t*-test	Only 12% of patients had satisfactory knowledge about glaucoma before the educational program compared to 96% after the program, with patients also having fewer incorrect beliefs about disease-causing factors as well as a better expectation and attitude toward their care and future health. Between 64 and 94% of patients also improved their practices of glaucoma eye care. Patients experienced fewer difficulties related to daily living skills, less anxiety, less depression, and higher self-esteem after the educational program
Moreno at al. (2018) Spain	Evaluate FV and QoL of children with glaucoma. Compare children’s QoL as reported by themselves and by their parents	Cross-sectional (questionnaire) FV, glaucoma-related QoL	24 children with glaucoma and their parents	4–16 years; *M* = 9.13 years	GQL-15 VFQ-25 Self-report and parent report	Wilcoxon test, correlations	Worse VR-QoL: worse VA in the best eye, increased visual field mean defect in the best eye; Not associated with child’s age, age at diagnosis, number of surgeries, number of clinic visits in past two years, months since last surgery, and number of eye drops; caregivers reported worse QoL of their children than children themselves Worse FV: visual field mean defect in the best eye was negatively associated with near activities, far activities, social function, dependency, and peripheral vision subscales
Silva et al. (2021) Brazil	Identify psychosocial indicators and assess the impact of filtering surgeries on QoL of children with PCG and their families	Cross-sectional (questionnaire and interview) PF, VR-QoL	9 children with bilateral PCG and their parents	17–65 months; *M* = 35 months	CVFQ Semi-structured interviews Parent-report	Cronbach’s alpha, correlations	Psychological inquiry: most frequently mentioned psychosocial indicators were disease knowledge, mother’s and family’s feelings on facing the surgical treatment, treatment adherence, social support, and future expectations Worse VR-QoL: worse VA in the best eye; competence subscale was negatively associated with axial length, and corneal diameter; treatment subscale was negatively associated with IOP, optic disk cupping, and corneal diameter; not associated with number of surgeries and number of eye drops
Zhang et al. (2009) China	Evaluate QoL scale for patients with PCG following antiglaucoma surgery at the last follow-up visit	Cross-sectional (questionnaire) glaucoma-related QoL	51 children and young people with PCG; 50 children and young people with normal vision	5–20 years; *M* = 7.8 years	PCG-QoL Self-report	Correlations, RA	Worse VR-QoL: patients with PCG had worse QOL compared to normal individuals; patients with PCG with more severe glaucoma, worse postoperative VA, and unsuccessful surgery reported worse overall QoL; no differences between gender and age groups Multiple-factor stepwise RA: patients who were introverts, had more severe glaucoma, and unsuccessful surgery reported worse QoL

*ANOVA* analysis of variance, *BCVA* best-corrected visual acuity, *CDI* Children’s Depression Inventory, *CMAS* Children’s Manifest Anxiety Scale, *CVAQC* Cardiff Visual Ability Questionnaire for Children, *CVFQ* Children’s Visual Function Questionnaire, *FV* functional vision, *GQL-15* Glaucoma Quality of Life, *HR-QoL* health-related quality of life, *IVI-C* Impact of Vision Impairment for Children, *Kidscreen-27* HR-QoL Questionnaire for Children and Adolescents, *LoA* Limits of Agreement, *LVP-FVQ-II* LV Prasad Functional Vision Questionnaire-II, *M* mean average, *PCG* primary congenital glaucoma, *PCG-QoL* Primary Congenital Glaucoma Quality of Life Scale, *PedsQL* Pediatric Quality of Life Inventory, *PROM* patient-reported outcome measure, *QoL* quality of life, *RA* regression analysis, *SCG* secondary childhood glaucoma, *SEI* Self-Esteem Index, *SWL* satisfaction with life, *SWLS* Satisfaction with Life Score, *VA* visual acuity, *VFQ-25* Visual Functional Questionnaire, *VI* visual impairment, *VR-QoL* vision-related quality of life, *WHOQOL-BREF* World Health Organization QoL Instrument – Abbreviated Version.

**Table 2:** Correlates and predictors of psychosocial functioning and quality of life of patients with childhood glaucoma.

Factors examined in relation to patients’ PF or QoL	Significant findings (*p* < 0.05)	Non-significant findings (*p* ≥ 0.05)
Clinical factors		
Worse visual acuity in the better eye	Worse FV [36, 38, 77] Worse glaucoma-related QoL [75, 79] Worse HR-QoL [36] Worse VR-QoL [36, 38, 77, 78, 82]	No difference in HR-QoL [37], QoL [90], and VR-QoL [81] No influence on the level of concordance between self- and parental reporting of HR-QoL [76]
Increase of visual field mean defect	Worse glaucoma-related QoL [79] Worse VR-QoL [81]	No difference in QoL [90]
Higher IOP		No difference in QoL [90] and VR-QoL [81, 82]
Type of glaucoma	Group with SCG had worse FV and VR-QoL than group with PCG [38] Group with PCG had worse glaucoma-related QoL than group with normal vision [75]	No difference in FV [77], HR-QoL [37], and VR-QoL [77]
Bilateral glaucoma (vs. unilateral)	Worse FV [36, 38, 77] Worse VR-QoL [77]	No difference in QoL [90], HR-QoL [36, 37], and VR-QoL [36, 38, 78] No influence on the level of concordance between self- and parental reporting of HR-QoL [76]
Higher number of surgical procedures	Worse FV [38, 77] Worse VR-QoL [77]	No difference in QoL [90], glaucoma-related QoL [79], HR-QoL [36], and VR-QoL [38, 78, 81, 82]
Longer duration since surgery		No difference in glaucoma-related QoL [79], and HR-QoL [37]
Higher number of clinical visits last year		No difference in glaucoma-related QoL [79]
Unsuccessful surgery (vs. successful surgery)	Worse glaucoma-related QoL [87]	
Higher number or frequency of glaucoma medication or treatment (e.g., higher number of eye drops)	Worse FV [77]	No difference in QoL [90], glaucoma-QoL [79, 81], HR-QoL [36], and VR-QoL [77, 78, 82]
Younger age at diagnosis/longer duration of glaucoma		No difference in FV [38, 77], glaucoma-related QoL [79], and VR-QoL [38, 77] No influence on the level of concordance between self- and parental reporting of HR-QoL [76]
Family with more than one child with glaucoma		No difference in FV [77] No influence on the level of concordance between self- and parental reporting of HR-QoL [76]
Sociodemographic factors		
Younger age of patients	Worse FV [36] Worse HR-QoL [37] Worse VR-QoL [36] Self-reported worse HR-QoL compared to parent report [76]	No difference in FV [38, 77], glaucoma-QoL [75, 79], HR-QoL [36], QoL [90], and VR-QoL [38, 77, 78, 81]
Female (vs. male)	Self-reported worse HR-QoL compared to parent report [76]	No difference in FV [38, 77], glaucoma-related QoL [75], HR-QoL [37], QoL [90], and VR-QoL [38, 77, 78]
Ethnicity group		No difference in VR-QoL [78]
Lower patients’ education level	Worse QoL [90]	
Lower caregivers’ education level	Worse VR-QoL [77]	No difference in FV [77] No influence on the level of concordance between self- and parental reporting of HR-QoL [76]
Parental age		No influence on the level of concordance between self- and parental reporting of HR-QoL [76]
Urban place of residency (vs. rural)	Worse QoL [90]	
Married (vs. single)		No difference in QoL [90]
Lower socioeconomic status		No difference in QoL [90]
Other factors		
Introverts (vs. extroverts)	Worse glaucoma-related QoL [87]	

#### Psychosocial functioning and quality of life of individuals with childhood glaucoma: associated clinical, management, treatment, and glaucoma-specific factors

The majority of studies reported possible differences in patient’s QoL and FV in relation to their VA. The results across the studies measuring VR-QoL were mostly consistent, with seven papers reporting worse QoL in cases of increasing visual field deviation [79, 81], worse VA [36, 38, 77–79, 82], or worse postoperative VA [75]. Though, one study found the association with VA not to be important [81]. Conversely, studies measuring HR-QoL [37] or generic QoL and life satisfaction [90] did not find their association with the level of VA and visual field mean deviation to be significant. Similar to VR-QoL, worse FV was also related to worse VA [36, 38, 77].

As for glaucoma factors, some studies found children with bilateral glaucoma to have significantly worse VR-QoL [77], HR-QoL [36], as well as satisfaction with life [90] and FV [36, 38] compared to children with unilateral glaucoma. One study [38] also found laterality to be a significant independent predictor of FV scores. Five papers reported laterality not to be associated with VR-QoL [36, 38, 78], HR-QoL [37], or generic QoL [90]. Type of glaucoma did not play an important role in QoL or in FV [77] in most cases [37, 75, 77]. One study observed that children with secondary childhood glaucoma reported significantly worse VR-QoL and FV than children with PCG despite comparable VA [38].

Children’s age at diagnosis or duration since diagnosis did not have a significant impact on either FV [38, 77] or QoL [38, 77, 79]. QoL was not associated with age at surgery [90] or time since the last surgery [37, 79], though children reported better self-care scores after a successful surgery compared to children with an unsuccessful surgery [75]. Most studies did not find a connection between number of glaucoma surgeries and patient QoL [38, 78, 79, 81, 82], except for one study that reported worse VR-QoL in children who underwent three or more surgeries [77]. Two studies observed poor FV (self-reported difficulty to complete an activity due to vision) in children who underwent one or more surgeries [38] and more than three surgeries [77]. Consistently, other types of medical treatment or application of medication (e.g., eye drops, antiglaucoma medication, number of clinic visits) were not associated with QoL in children with glaucoma [36, 77–79, 81, 82]. With regard to FV, one study observed that children who required less daily eye drops had better FV scores [77].

Glaucoma knowledge and expectations of eye care significantly impact family’s PF and well-being [71, 82]. In one study [71], an educational program on glaucoma resulted in participants reporting more accurate knowledge about glaucoma and disease causes. Patients also improved their attitudes and practices toward glaucoma care and future health. Importantly, participants demonstrated significantly fewer difficulties with activities of daily living, higher self-esteem, and improved mental health. In another study [82], patient and family QoL factors were evaluated. Significant psychosocial indicators included knowledge of glaucoma, treatment adherence, present social support, future expectations from the point of optimism, and uncertainty surrounding the child’s diagnosis.

#### Psychosocial functioning and quality of life of individuals with childhood glaucoma: associated sociodemographic factors and characteristics of patient and family

Studies assessed the relationship between PF and QoL measures and sociodemographic factors, such as age, gender, and ethnicity. The majority of these studies did not find age or gender to be associated with QoL [36–38, 75, 77–79, 90] or FV scores [36, 38]. However, one study found patients of young age (8–11 years old) and female gender to report worse HR-QoL and showed greater disagreement with parental reports than adolescent and male patients, respectively [76]. Only one study looked at potential ethnicity-related differences, but did not find an association with VR-QoL scores [78]. In a study on young adults with childhood glaucoma [90], married adults reported higher life satisfaction, and marital status explained 8.8% of the variance in the participants’ life satisfaction scores. The authors also found that adults living in rural environments and with higher education levels reported better overall QoL, together explaining the 13% of the variance in the participants’ QoL scores. Interestingly, the socioeconomic status of these participants did not play a role in their QoL or life satisfaction. Parental education level was not associated with children’s VR-QoL or FV [77]. A cross-sectional study measuring personality characteristics found introverted children to report significantly lower scores on social and mental domains of VR-QoL compared to those of extroverted children [75]. However, it is important to note that the authors did not specify how these personality traits were measured and categorized in the two groups.

### The impact of childhood glaucoma on caregivers

The summary of studies on caregivers to patients with childhood glaucoma is outlined in Table 3 and correlates and predictors of QoL and/or FV for those caregivers are presented in Table 4. Caregiver outcomes related to PF were measured as mental health, caregiver burden, positive aspects of caregiving, and QoL, with most studies also assessing their relationship to different clinical and sociodemographic factors.

**Table 3:** Summary of the studies on caregivers of patients with childhood glaucoma.

Author, publication year, and country	Study aim	Study type and area of interest	Sample	Patients’ and caregivers’ age range; average age	Instruments and reporter	Data analysis	Main results
AlQurashi et al. (2019) Saudi Arabia	Assess QoL of caregivers of children with glaucoma	Cross-sectional (questionnaire) Caregivers’ QoL	85 caregivers of children with glaucoma	Caregivers: 26–62 years, *M* = 42.5 years; children: *M* = 12.9 years	CarCGQoL Self-report	Linear RA	Multiple linear RA: caregivers who were mothers, currently unemployed/retired, with ≥1 child with glaucoma, and caring for children with worse VA reported worse QoL (*R*^2^ = 22.3%); not associated with marital status, caregivers’ formal education, child’s gender, age of diagnosis, laterality of glaucoma, and surgical interventions
Dada et al. (2013) India	Evaluate the level of caregiver burden and depression in primary caregivers of children with PCG	Cross-sectional (questionnaire) Caregivers’ burden, Caregivers’ mental health	55 primary caregivers of children with PCG	Caregivers: 20–42 years, *M* = 23.6 years; children: 1–36 months, *M* = 8.1 months	CBQ PHQ-9 Self-report	Percentages, one-way ANOVA, Wilcoxon rank-sum test, post hoc analysis (Bonferroni test, Kruskal–Wallis test)	Caregiver burden and depression: higher scores of socioeconomic, emotional, and aggregate burden were related to higher severity of depression
Gothwal et al. (2015) India	Develop and validate CarCGQoL questionnaire	Cross-sectional (questionnaire) Development and validation	111 caregivers of children with PCG	Caregivers: 18–41 years, *M* = 25.3 years; children: *M* = 5.7 months	CarCGQoL Self-report	Rasch analysis	The instrument was deemed misfit by the Rasch model and lacked a unidimensional structure. The number of items was reduced from 45 to 20, resulting in good fit and unidimensional structure
Gothwal et al. (2016) India	Assess the changes in QoL of caregivers of children with PCG before and after glaucoma surgery	Longitudinal (questionnaire) Caregivers’ QoL	111 caregivers of children with PCG	Caregivers: 18–41 years, *M* = 25.3 years; children: *M* = 5.7 months	CarCGQoL Self-report	Logistic regression	QoL: better postoperatively compared to baseline; the improvement in their QoL (by >2-fold) was associated with their child’s surgical procedure at 6–8 weeks postoperatively; not associated with child’s surgery outcome nor any sociodemographic characteristics
Gothwal et al. (2020) India	Examine cross-diagnostic validity of CarCGQoL questionnaire among caregivers of children with congenital cataract, retinopathy of prematurity, and blinding corneal disorders	Cross-sectional (questionnaire) Validation	891 caregivers of congenital cataract, ROP, blinding corneal disorders + reference group (PCG)	Caregivers: *M* = 28.3 years; children: *M* = 36.3 months	CarCGQoL Self-report	Rasch analysis	Six items were removed across three groups. The questionnaire showed acceptable measurement reliability. It showed a unidimensional structure but with some differential item functioning
Kantipuly et al. (2019) India	Identify sociodemographic and clinical factors associated with QoL in caregivers of children with PCG in south India	Cross-sectional (questionnaire) Caregivers’ mental health, caregivers’ QoL	70 caregivers of children with PCG	Caregivers: *M* = 32.1 years; children: 1.5–18 years, *M* = 7.7 years	PHQ-9 CarCGQoL Self-report	Rasch analysis, *t*-test, *χ*^2^ test, linear RA, correlations	Worse QoL: decreasing child’s age, longer duration of glaucoma, more depression symptoms; not associated with gender, caregiver’s age, caregiver’s education, SES, and number of surgeries Depression: no other variable but QoL was associated with the level of depression
Knight et al. (2022) Australia and New Zealand	Investigate the impact of childhood glaucoma on the caregivers and their QoL	Qualitative (interview) PF and QoL	35 caregivers of children with glaucoma	Caregivers: *M* = 50.2 years	Semi-structured interviews Self-report	Interpretive phenomenology	6 major QoL themes found: coping, emotional well-being, medical and social support, social well-being, clinical and familial control, family planning
Wy et al. (2022) Korea	Develop and validate a decision tree model to identify caregivers of children with glaucoma at risk for anxiety and depression	Cross-sectional (development of decision tree model) Caregivers’ mental health	129 caregivers of children with glaucoma	Caregivers: NS children: 0–18 years	DHS PHQ-9 GAD-7 Self-report	One-way ANOVA, decision tree analysis	Anxiety and depression: no differences between caregivers of children with PCG, SCG, or asymptomatic glaucoma suspect Decision tree analysis: higher number of glaucoma surgeries and worse VA in better eye were identified as decision nodes determinative of caregivers’ moderate-to-severe depressive symptoms as well as moderate-to-severe anxiety symptoms
Zhu et al. (2019) China	Assess the burden and positive aspects of caregiving for patients with pediatric glaucoma and identify characteristics of the caregivers and the patients related to them	Cross-sectional (questionnaire) Caregivers’ burden, positive aspects of caregiving	57 caregivers of children with glaucoma	Caregivers: *M* = 30.02 years; children: *M* = 30.09 months	CBI PAC Self-report	Linear RA	Caregiver burden and PAC: emotional burden was negatively associated with aggregated PAC and outlook on life subscale Linear RA: mothers, more educated, with higher household income, and with a child with longer disease duration reported more caregiver burden; caregiver burden was not associated with number of surgeries; PAC scores were not associated with any of the demographic factors

*ANOVA* analysis of variance, *CarCGQoL* Caregiver’s Congenital Glaucoma Quality of Life, *CBI* Caregiver Burden Inventory, *CBQ* Caregiver Burden Questionnaire, *DHS* Demographic and Health Survey, *GAD-7* Generalized Anxiety Disorder, *LR* linear regression, *M* mean average, *NS* not specified, *PAC* Positive Aspects of Caregiving, *PCG* primary congenital glaucoma, *PHQ-9* Patient Health Questionnaire, *QoL* quality of life, *RA* regression analysis, *SCG* secondary childhood glaucoma, *VA* visual acuity.

**Table 4:** Correlates and predictors of psychosocial functioning and quality of life of caregivers of patients with childhood glaucoma.

Factors examined in relation to caregivers’ PF or QoL	Significant findings (*p* < 0.05)	Non-significant findings (*p* ≥ 0.05)
Clinical factors		
Worse visual acuity in the better eye	More anxiety and depression symptoms [88] Worse QoL [83]	
Higher IOP		No difference in anxiety and depression symptoms [88]
Type of glaucoma		No difference in anxiety and depression symptoms [88] and QoL [83]
Laterality of glaucoma		No difference in anxiety and depression symptoms [88] and QoL [83]
Higher number of surgical procedures	More anxiety and depression symptoms [88]	No difference in caregiver burden [87], depression symptoms [86], positive aspects of caregiving [87], and QoL [86]
Postoperative change and surgery success	Better QoL after the surgery, regardless of the surgery’s success [85]	
Age at surgery		No difference in QoL [85]
Number or frequency of glaucoma medication or treatment (e.g., number of eye drops)		No difference in anxiety and depression symptoms [88]
Younger age at diagnosis/longer duration of glaucoma	More caregiver burden [87] Worse QoL [86]	No difference in depression symptoms [86], positive aspects of caregiving [87], and QoL [83]
Family with more than one child with glaucoma	Worse QoL [83]	
Sociodemographic factors		
Caregiver’s age		No difference in anxiety [88] and depression symptoms [86, 88], caregiver burden [87], positive aspects of caregiving [87], and QoL [85, 86]
Female caregiver (vs. male)	More caregiver burden [87] Worse QoL [83]	No difference in anxiety [88] and depression symptoms [86, 88], positive aspects of caregiving [87], and QoL [85, 86]
Child’s gender		No difference in caregiver burden [87], positive aspects of caregiving [87], and QoL [83, 85]
Younger age of the child	Worse QoL [86]	No difference in caregiver burden [87], depression symptoms [86], and positive aspects of caregiving [87]
Lower parental education level	Less caregiver burden [87]	No difference in depression symptoms [86], positive aspects of caregiving [87], and QoL [83, 85, 86]
Unemployed (vs. employed)	Worse QoL [83] Less caregiver burden [87]	No difference in positive aspects of caregiving [87] and QoL [85]
Married (vs. single)		No difference in QoL [83]
Lower socioeconomic status	Less caregiver burden [87]	No difference in depression symptoms [86], positive aspects of caregiving [87], and QoL [85, 86]
Other factors		
More depressive symptoms	More caregiver burden [84] Worse QoL [86]	

#### Psychosocial functioning and quality of life of caregivers of individuals with childhood glaucoma: the impact of the diagnosis

In contrast to the results of studies focusing on the patient, caregiver studies reported few significant associations between PF or QoL and glaucoma-specific factors. The child’s age at diagnosis was not significantly associated with caregiver QoL [83, 86, 88]; however, caregivers whose child had a longer duration of glaucoma reported significantly worse QoL [86] and more time-dependent and overall burden [87] compared to caregivers of children with more recent diagnoses. Furthermore, those caregivers with children with worse best-corrected visual acuity (BCVA) reported significantly lower QoL [83] and more anxiety and depression symptoms [88] compared to that of caregivers of children with better BCVA. Caregivers were also at higher risk for moderate-to-severe anxiety and depression if their child had undergone a surgical procedure [88]. Interestingly, one study found improved caregiver QoL following their child’s surgical procedure, regardless of the surgical outcome [85]. Three studies did not find the number of surgeries to have an impact on caregiver QoL [83, 86, 87]. The strongest negative predictor of caregiver QoL in one study was the number of additional children with glaucoma [83], though a separate study [85] found no association between additional children with glaucoma and change in caregiver QoL before and after their child underwent surgery. Other glaucoma-related factors such as age at surgery [85], glaucoma medication [83, 88], laterality [83, 85], type of glaucoma [83, 88], and PCG severity [85] were not associated with caregiver QoL, mental health, or the level of caregiver burden.

#### Psychosocial functioning and quality of life of caregivers of individuals with childhood glaucoma: associated sociodemographic, caregiver, and family characteristics

One study found that 20% of caregivers reported anxiety symptoms, with 8.5% experiencing moderate-to-severe anxiety [88]. Depression or depressive symptoms were highly prevalent among caregivers of children with glaucoma and were negatively associated with caregiver QoL [86]. The prevalence of depressive symptoms among caregivers varied across studies (23.6% [88], 44% [86], and 69.1% [84]), which may be explained by unaccounted transient situations. For example, one study revealed that 71% of caregivers demonstrated poor to very poor pre-operative QoL with associated agitation, irritability, depression, anxiety and powerlessness, though this prevalence dropped to 20% post-operation [85].

Not surprisingly, emotional, socioeconomic and overall burdens likewise increased with the severity of depression [84]. Between 47.4 and 71% of caregivers experienced a moderate burden, and between 5% and 8.8% of caregivers felt severely burdened from caring for children with glaucoma [84, 86].

In another study, outcomes of caregiving were measured using a questionnaire that ranged from levels of positive aspects to levels of the burden of caregiving and assessed self–affirmation (e.g., increasing meaning to life) and outlook on life (e.g., positive attitude). Although a majority of caregivers showed some level of burden, most reported a moderate level of positive aspects of caregiving for children with glaucoma [87]. Only emotional burden was negatively related to the overall positive aspects of caregiving, indicating caregivers whose children behave more unpredictably were also more likely to feel unappreciated and less useful within the context of caregiving. Sociodemographic or family factors did not play an important role in the positive experience of caregiving [87].

Most studies did not find QoL to be related to caregiver sociodemographic characteristics, such as age [85, 86], gender [85, 86], marital status [83, 85], socioeconomic status, average household income [85, 86], or employment status [83, 85]. Similarly, most studies found child gender [83, 85, 86, 88] and age [83] unrelated to caregiver QoL and mental health, though caregivers of older children with PCG reported worse QoL compared to caregivers of younger children with PCG in one study [86].

These characteristics played an important role in caregiver burden in a study by Zhu et al. [87]. Overall caregiver burden was significantly higher in mothers, caregivers with higher education or household income, and employed caregivers. More specifically, parents who were older, had lower household income, or whose children were younger experienced a more time-dependent burden. The same authors also described that mothers and employed caregivers experienced more physical burden, while less-educated caregivers reported more developmental burden [87].

### Study designs of included publications

The ideal study design for assessing PF or QoL in childhood glaucoma would have a large sample size, use an internationally validated instrument specific to childhood glaucoma, address each facet of PF or QoL individually, and be designed longitudinally. Currently, there are no studies that meet all the criteria outlined above.

The existing literature on PF or QoL in childhood glaucoma mainly comprises cross-sectional studies. Only two longitudinal studies were conducted, one in India studying caregivers [73] and the other in Egypt studying children [71]. Few studies reported sample size calculations, but those that did meet [86] or exceed [83] their target enrollment were determined using expected impact and prior literature.

**Fig. 2:**
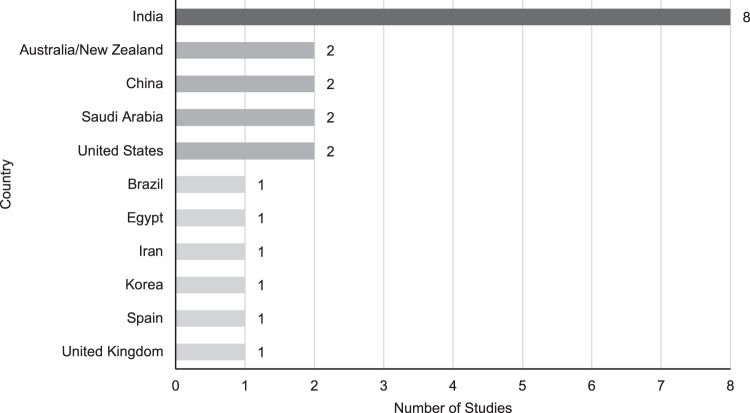
Geographic distribution of literature. The y-axis includes the countries where studies were conducted, and the x-axis tallies the number of studies produced per country. India conducted the highest number of studies relevant to this topic, with 8 total papers included in this review.

Most studies addressed the PF or QoL of children diagnosed with childhood glaucoma, while three studies included adults [80, 81, 90] with a glaucoma diagnosis at pediatric age. One study was considered for review but excluded due to the participants’ age at diagnosis (15–40 years), which did not fit the authors’ definition of childhood glaucoma [91]. Geographically, a disproportionate number of studies was conducted in India (Fig. 2), with one study group contributing seven of the studies.

Only eight of the studies included in the review assessed elements of PF. Of those, four studied only PF [71, 84, 87, 88], and four measured both PF and QoL [80, 82, 86, 89]. Notably, Knight et al. studied PF and QoL as congruent topics encompassing emotional well-being, social well-being, inconveniences, activity limitations, and mobility in adults with a childhood glaucoma diagnosis [80] and in caregivers [89]. The remaining 14 discussed only QoL, independent of PF. No studies assessed the burden of childhood glaucoma on family members other than parents, such as siblings, or on providers who manage childhood glaucoma. Findings on PF or QoL in childhood glaucoma across the published studies remain inconsistent due in part to the cultural diversity among study populations, which limits generalizability of the findings to a global scale.

### Variability of tools and results

Twenty-one instruments were used among the 22 included papers (Table 5), demonstrating a lack of consistency or standardization when researchers considered how to assess PF or QoL in childhood glaucoma. Instruments used for patient-reported outcomes measured VR-QoL, HR-QoL, general QoL, and FV, and were catered to populations with visual impairment [78] or pediatric populations in general (i.e., PedsQL or Kidscreen-27). Caregiver PF or QoL was assessed using a questionnaire specific to childhood glaucoma caregiving (CarCGQoL) as well as general caregiver instruments (i.e., PAC, CBQ, CBI). For both populations, commonly used mental health instruments included PHQ-9 or GAD-7. Most studies also incorporated supplemental questionnaires recording sociodemographic data and ocular medical history. The variability in the instruments was likely a contributing factor to the variability in results.

**Table 5:** Instruments used in literature on PF or QoL in childhood glaucoma for patients and caregivers.

Instrument	Frequency in review
Cardiff Visual Ability Questionnaire for Children (CVAQC)	2
Caregiver’s Congenital Glaucoma Quality of Life (CarCGQoL)	5
Caregiver Burden Index (CBI)	1
Caregiver Burden Questionnaire (CBQ)	1
Children Depression Inventory (CDI)	1
Children Manifest Anxiety Scale (CMAS)	1
Children’s Visual Function Questionnaire (CVFQ)	1
Generalized Anxiety Disorder (GAD-7) Assessment	1
Glaucoma Quality of Life (GQL-15)	1
Impact of Vision Impairment for Children (IVI-C)	4
Kidscreen-27	2
LV Prasad Functional Vision Questionnaire-II (LVP-FVQ-II)	1
Positive Aspects of Caregiving (PAC)	1
Patients’ Expectations Scale	1
Primary Congenital Glaucoma Quality of Life Scale (PCG-QoL)	1
Pediatric Quality of Life Inventory (PedsQL)	1
Patient Health Questionnaire (PHQ-9)	3
Self-Esteem Inventory	1
Satisfaction with Life Score (SWLS)	1
Visual Functional Questionnaire (VFQ-25)	2
World Health Organization QoL Instrument – Abbreviated Version (WHOQOL-BREF)	1

While a universal tool to assess PF or QoL may prove useful in drawing more generalizable conclusions, researchers developing, translating, and validating an instrument for use across various regions must consider language and culture. Culture often plays a large part in how ideas are communicated. Particularly for rural populations, variability in local colloquialism can change the meaning of a question entirely, making direct translation challenging. Culture can also dictate standards of well-being that may not be applicable worldwide [73]. For example, an instrument developed in India includes “likelihood that child will get married” [73], which, while culturally significant in India, may not universally apply.

The specificity of instruments developed can affect the accuracy of reported PF or QoL. It is important to have an instrument that is disease (glaucoma) specific in order to capture the unique challenges that the chronic condition presents, i.e., the specific impact of an uncertain visual prognosis and potentially blinding progressive disease; yet it will unsurprisingly not be applicable to populations without the disease [36]. Conversely, if an instrument is generic, it will be able to reach a broader population, but may not properly evaluate characteristics distinct to the disease(s) studied.

Furthermore, distinct concepts such as VR-QoL and FV have been inappropriately conflated in some ophthalmic literature [44]. While FV might be related to VR-QoL, they are operationalized differently. For example, FV does not include mental health, self-perception, or social functioning and is restricted to vision-related activities (i.e., the ability to watch TV), whereas VR-QoL is the patients’ perception of their own lives in relation to their eye problem (i.e., ability to make friends). The authors encourage the use of unidimensional instruments that do not conflate distinct concepts.

Another challenge to measuring PF or QoL in childhood glaucoma involves the perception and reporting of PF or QoL itself. The age at which children can reliably and accurately self-report results is inconsistent. Some studies have validated self-reporting by children as young as 5 years old [92], while other literature states children cannot reliably self-report until the age of 8 [93]. Furthermore, studies that compare children’s self-reported PF, QoL, or FV to those reported by caregivers found that caregivers consistently overestimate the burden of disease on children and underestimate their QoL [36, 79, 94], apart from one study, which showed bidirectional disagreement between patients and their caregivers (i.e., caregivers both over- and underestimated their child’s QoL) [76]. These limitations make it difficult to determine whether QoL reports by younger children or by proxy caregivers are reliable.

## Discussion

### Strengths

This is a novel, childhood glaucoma-specific comprehensive review of PF and QoL in children and caregivers. While there is a breadth of literature concerning QoL in patients with visual impairment and their caregivers [1–4, 6, 8, 9, 67], no extant review exists specific to childhood glaucoma.

A thorough and comprehensive search of all potentially relevant studies was conducted (details on this may be found in the section titled “Method of literature search”). Due to the paucity of research overall, literature published in any year was included. As a result, we believe that this review encompasses all literature published on PF or QoL and childhood glaucoma with either an abstract or text available in English. Still, there is a chance that relevant studies may have not been included in this review. Our results, however, indicate that investigations on this topic only recently began. Of the 22 studies included in the current review, the earliest was published in 2009, and the majority (*n* = 16) appeared in the literature in the last 5 years.

PROMs can help healthcare professionals to make informed decisions based on their patients’ priorities and improve the care they provide as well as evaluate the performance of their practice [15]. This review demonstrates the utility of PROMs, like the PF or QoL assessment tools, to monitor the progress of patients with childhood glaucoma, measure the impact of healthcare interventions, detect secondary problems early, and measure caregiver burden.

Lastly, this review identifies questionnaires that can be employed in clinical practice to assess childhood glaucoma’s impact on patient and caregiver health outcomes.

### Limitations

There are a number of methodological issues that limit the quality of the studies reviewed. The foremost limitation of this review is that there are few definitive conclusions that can be made from the current evidence base, which is likely due to the quality of the studies present, such as small sample sizes and selective patient demographics. As discussed, the lack of standardized questionnaires specific to PF or QoL in childhood glaucoma led to researchers using several different instruments or adapting them to the population of interest, thus impacting the ability to capture glaucoma-specific concerns or impacts. Similarly, the PROMs implemented in some studies of our review do not differentiate between QoL and FV as distinct concepts, leading to imprecise results [44]. Furthermore, the literature disproportionately represents study samples from India or the Middle East, with relatively little research conducted in other parts of the world (Fig. 2), which could be an outcome of the variation in the prevalence of childhood glaucoma per global region. Cultural factors as well as variations in study methods and samples may also limit the applicability of the findings to population which differ substantially.

The authors included literature on any methodology, a potential cause for the discrepancy among the results. Two studies focused specifically on questionnaire development and validation [73, 74] and one study measured the parent–child agreement in reporting HR-QoL [76]. Two studies featured a longitudinal study design [71, 85], one of which introduced an educational intervention [71]. The remaining 17 employed a cross-sectional study design, which cannot infer causality and does not address how PF or QoL may change for patients and their caregivers over time. In addition, many studies did not utilize a control group but rather compared results to the norms of the various measures used. Another limitation was the relatively small sample sizes employed in several studies. There is also the question of possible study sample bias in many studies which did not report participation rates. Two sets of papers [37, 73, 76, 85] derived their results from the same dataset, potentially duplicating findings.

While a number of studies examined QoL in young patients with glaucoma, there were no studies of psychological and social functioning, cognitive development, or academic achievement. Among studies of QoL, few examined generic as opposed to vision-specific QoL. More studies are needed to address factors predictive of QoL (besides visual ability, bilateral condition, number of surgeries) as well as interventions to improve QoL. As the research literature in this area increases, the use of meta-analysis will be a useful tool to compare results across various studies as well as systematically evaluate the rigor of the methodologies employed. Prospective longitudinal studies with large and diverse patient samples [95] are especially needed to identify the course of psychosocial development and factors predictive of health outcomes.

### Opportunities for global and public health research

Future prospective, longitudinal studies would be useful in understanding changes in PF or QoL over time as it relates to factors such as age, length of therapy, nature of the intervention, visual outcome, and complications, when applicable. As previously discussed, the development of globally accepted standard instruments for measuring PF or QoL may aid in comparing results across regions, while also appreciating how local population-specific factors might affect these. The authors also encourage a global collaboration to diversify the sample population and study size in future research.

Studying the correlations between PF or QoL and factors such as education level, health literacy, age, and marital status may improve our understanding of the effect of glaucoma on an entire family. Research to elucidate this complex area and identify interventions to support families is warranted.

The authors note that while many of the studies and interventions discussed in this review reported or addressed reports of childhood glaucoma having a negative impact on the activities of daily living of the patient and caregiver, this does not necessarily translate into lower QoL. The “disability paradox” indicates that a child’s QoL is not inherently dependent on whether or not they have functional limitations or health conditions, and therefore children with disabilities may experience QoL that is as good as, or sometimes better than, their non-disabled peers [14]. It would be of interest to investigate these issues in childhood glaucoma in diverse populations and cultures. Age and developmentally appropriate PROMs that measure QoL and FV separately are also necessary to identify the disability paradox in glaucoma populations should it exist.

### Opportunities for clinic-based research

This review highlights the void as well as the potential value of using standardized outcome measures to monitor PF or QoL in patients and caregivers. Indeed, some studies on utilizing PROMs in routine clinical care display a positive impact on HR-QoL [96, 97]. Given the tendency for caregivers to under- [36, 76, 79] or overestimate [76] their child’s well-being, the authors advocate seeking self-report from subjects as the most important outcome measure rather than caregiver or proxy report when administering questionnaires related to PF or QoL in the patient. Caregivers are an integral part of the clinical relationship, and this needs to be respected when seeking such information. Raising awareness of this potential source of bias, along with specific comments on study design methodology to show how this has been addressed, will strengthen the veracity of the research findings. Furthermore, to comprehensively understand well-being in childhood glaucoma care, researchers should consider studies assessing PF or QoL in siblings and in those who provide care to individuals with childhood glaucoma. It would also be useful to explore the impact of the health provider’s or the siblings’ attitudes toward the child with glaucoma upon that child.

### Opportunities for interventional research

Multiple studies included in this review identified that low FV is associated with low VR-QoL and HR-QoL [36, 38, 75, 77, 79], revealing an extra incentive to focus efforts to maximize visual health. Low-vision aids, such as manual monocular telescopic systems, can improve children’s ability to succeed developmentally, socially, and academically [98], and maybe a necessary treatment for childhood glaucoma in addition to medical and surgical therapies [83]. Research on interventions outside of the aforementioned low-vision aids is limited, leaving an opportunity for future studies.

The accelerated application of telehealth during the COVID-19 pandemic has offered options for care, but evidence of its benefits and disadvantages is mixed. Telemedicine may improve patient experience, as seen by a decreased travel burden in one review on adult glaucoma care [99]. However, “digital exclusion” of patients who lack the resources to participate in telemedicine is a growing concern and a cause of widening health inequalities [100]. There are no current studies on this topic as it pertains to childhood glaucoma and thus, studies on the outcomes of telemedicine on PF or QoL in these individuals are recommended.

There is also no literature on the role of online support groups in improving well-being in individuals with childhood glaucoma or their caregivers, and this is an area of research that the authors believe could prove clinically useful. The low cost and potential for dissemination of information through online avenues allow for a greater scope of outreach, and online interventions can help deliver benefits to patients and caregivers globally.

It is fundamental to a successful assessment program that patients noted to have low PF or QoL have access to appropriate clinical resources and support structures. In addition to specialist glaucoma care, the clinical care team ideally should include members with skills in childhood and family behavior, psychology, low vision, occupational therapy, social work, and more. Literature also emphasizes the value of health literacy regarding illness uncertainty [71, 72] and the authors encourage providing psychoeducation to patients and caregivers to improve treatment, compliance, and family adaptation.

### Summary

This report addresses the peer-reviewed literature related to PF and QoL in patients with childhood glaucoma and their caregivers. The goal was to consolidate and describe the currently published evidence base, explore areas for relevant future research, and propose potential improvements in study design and methodology to deliver the highest quality evidence to improve health outcomes in this population.

Although some trends could be identified, such as significantly worse patient and caregiver PF or QoL with worse VA or younger patient age, the disparate approaches and study designs made more detailed analysis difficult. There were many inconsistencies at all stages of the study design, which while contributing to a broader picture, limit comparisons of outcomes between them, their relevance to other populations, or consolidation for meaningful collaborations and statistical analysis. Immediate important questions resulting from this work include how best to determine the most appropriate model(s) for assessment of the impact of childhood glaucoma in various healthcare settings, both for identifying patients and families who need additional support or other interventions, and how best to ensure that measures of patient and caregiver well-being are integral to these studies.

Our findings suggest healthcare teams will gain much from addressing the PF or QoL of childhood glaucoma patients and their caregivers as components of a holistic and multidisciplinary approach for families facing childhood glaucoma. The negative impact of childhood glaucoma extends into every domain of the life of the patient and beyond the patient in question to their caregivers. This impact cannot be captured through the clinical findings normally focused on by managing physicians. Whilst the literature is varied, a common theme is an advocacy for the inclusion of patient [36–38, 71, 75, 77, 78, 82, 90] and caregiver [73, 82–87] PF or QoL in the evaluation and treatment plan. Drawing from our review, potential avenues to address this need include discussions about health literacy [90], health educational intervention programs [71], and psychological support [75, 82, 84, 87]. There were relatively few common findings across the identified papers because of the wide variability in assessment tools, methodology, and cultures. This review highlights the need for future studies validating and employing standardized tools to measure PF or QoL of patients with childhood glaucoma and their caregivers.
